# Facile One-Pot Synthesis of Fe_3_O_4_ Nanoparticles Composited with Reduced Graphene Oxide as Fast-Chargeable Anode Material for Lithium-Ion Batteries

**DOI:** 10.3390/ma17205059

**Published:** 2024-10-17

**Authors:** Honggyu Seong, Taejung Jung, Sanghyeon Kim, Jaewon Choi

**Affiliations:** 1 Department of Chemistry and Research Institute of Molecular Alchemy, Gyeongsang National University, Jinju 52828, Republic of Korea; gu9188@gnu.ac.kr (H.S.); xajo1122@gnu.ac.kr (T.J.); 2 Department of Materials Science and Engineering, Inha University, Incheon 22212, Republic of Korea

**Keywords:** iron(III) oxide, reduced graphene oxide, Fe_3_O_4_-based composite materials, fast chargeable anode materials, lithium-ion batteries

## Abstract

To address the rapidly growing demand for high performance of lithium-ion batteries (LIBs), the development of high-capacity anode materials should focus on the practical perspective of a facile synthetic process. In this work, iron oxide nanoparticles (Fe_3_O_4_ NPs) in situ grown on the surface of reduced graphene oxide (rGO), denoted as Fe_3_O_4_ NPs@rGO, were prepared through a facile one-pot synthesis under the wet-colloidal conditions. The synthesized Fe_3_O_4_ NPs showed that uniform Fe_3_O_4_ NPs, with a size of around 9 nm, were distributed on the rGO surfaces. When applied as an anode material for LIBs, the Fe_3_O_4_ NPs@rGO anode revealed a high reversible capacity of 1191 mAh g^−1^ at 1.0 A g^−1^ after 200 cycles. It also exhibited excellent rate performance, achieving 608 mAh g^−1^ at a current density of 5.0 A g^−1^ over 500 cycles, with improved electronic and ionic conductivities due to the rGO template. This suggested that practically available anode materials can be developed through our one-pot synthesis by in situ growing the Fe_3_O_4_ NPs.

## 1. Introduction

The demand for more efficient energy storage systems (ESS) has been rapidly increasing due to global restrictions on fossil fuels and the growing need for improved electric vehicle (EV) performance. Lithium-ion batteries (LIBs), one of the traditional types of ESS, have been used as portable power sources for various electrical devices as well as EVs, with their high energy densities over recent decades (e.g., around 250 Wh kg^−1^ for graphite‖lithium nickel manganese cobalt oxide (NMC) cells) [1]. However, as demand for a longer range of EVs has been continuously growing, the higher energy density of LIBs is called for, pushing the development of next-generation electrode materials. While the separator and electrolyte are important, the electrode materials play the most critical role in LIB performance, as they are responsible for the insertion and storage of Li^+^ ions. Therefore, it is essential to improve the energy storage of LIBs and find alternatives with reinforced charge–discharge cycle performance.

For the negative electrode, graphite, a commercial anode material, exhibits good cycle stability and low working voltage, but its poor theoretical capacity (372 mAh g^−1^) precludes commercial LIBs from having a high specific capacity. Thus, various materials, such as alloying-type or conversion-type materials, have been studied to be applied as new anode materials for LIBs. Fe_3_O_4_, as one of the conversion-type materials, reacts with Li^+^ ions by producing a reduced metallic Fe^0^ and Li_2_O through the conversion reaction of transition metal oxides when storing Li^+^ ions (Equation (1)) [2].

(1) $$M_{x}O_{y}+\left(2\cdot y\right){Li}^{+}+\left(2\cdot y\right)e^{-}\to xM^{0}+y{Li}_{2}O$$

Among the conversion-type metal oxides, the Fe_3_O_4_-based materials have some advantages, such as a high theoretical capacity of 926 mAh g^−1^ and cheap iron ore price at US $102.83 per metric ton as of 1st August 2024 (Market Index Iron Ore. n.d.) [3]. These benefits have led many researchers to explore Fe_3_O_4_-based anode materials with a high specific capacity, cycle stability, and rate capability. Recently, the Fe_3_O_4_-based composite structures with carbonaceous materials have extensively been reported as a method to dramatically improve their cycle performance by stabilizing Fe_3_O_4_-based materials and reinforcing their electrochemical properties [4,5,6,7,8,9]. For example, Gao et al. synthesized yolk-shell porous Fe_3_O_4_@C anchored on graphene nanosheets (Y-S-P-Fe_3_O_4_/GNs@C), which exhibited excellent cycling stability with 579 mAh g^−1^ at a current density of 2.0 A g^−1^ over 1800 cycles [10]. Similarly, Xie et al. reported hollow Fe_3_O_4_/rGO composites (H-Fe_3_O_4_/rGO). It displayed a specific capacity of 827.3 mAh g^−1^ at a current density of 0.5 A g^−1^ after 550 cycles [11].

Many previous works, such as the above examples, presented various advanced composite structures by introducing modified carbonaceous materials. However, their methods were not simple and practical for commercialization due to two or more cumbersome synthetic processes. Therefore, next-generation anode materials should be further explored in the practical perspective for commercial LIBs. Herein, two strategies to approach the practical application of Fe_3_O_4_-based anode materials for LIBs with high cycling performance were adopted in this study. The first way is the use of iron(III) acetyl acetonate as a precursor for Fe_3_O_4_. It is well-known that the metal acetyl acetonate can be used as precursors for a conversion to metal oxides product [12]. Because metal acetyl acetonates already embed M–O bonds, they provide better atom-efficiency, serving as single precursors for the synthesis of the metal oxides through thermal decomposition, thereby minimizing the additives required for manufacturing. The second way is adding the commercial carbonaceous materials to the reaction pot for the synthesis metal oxides from metal acetyl acetonates. This method can make the seed of metal oxides to easily anchor on the surface of carbonaceous materials, which act as templates to easily afford composite structures.

Based on these strategies, this study suggests a facile synthetic pathway for an Fe_3_O_4_-based composite structure using a practical one-step process. Fe_3_O_4_ nanoparticles (NPs) were in situ grown on the surface of reduced graphene oxide (rGO) by simply heating an Fe(acac)_3_ precursor dissolved in oleylamine surfactant, denoted as Fe_3_O_4_ NPs@rGO. The as-prepared Fe_3_O_4_ NPs@rGO composite showed a promising potential as a fast-chargeable anode material, demonstrating discharge capacities of 1191 after 200 cycle and 608 mAh g^−1^ after 500 cycles at current densities of 1.0 and 5.0 A g^−1^, respectively. Notably, these excellent rate performance of Fe_3_O_4_ NPs@rGO anode, derived from the one-pot synthesis strategy, surpassed those of many reported Fe_3_O_4_-based anode materials prepared through complex synthetic processes. The reinforced rate performance was attributed to their improved electronic and ionic conductivities compared to bare Fe_3_O_4_ NPs anode. Therefore, this study provides great benefit to design and prepare practical alternatives to commercial anode materials for LIBs.

## 2. Materials and Methods

### 2.1. Materials

All chemicals were purchased from Merck (Darmstadt, Germany) and Alfa Aesar (Ward Hill, MA, USA) and used without further purification. Commercial rGO powder was purchased from Grapheneall Co., Ltd. (Hwaseong, Republic of Korea). Conductive carbon black (Super P from Timcal, Ltd. (Bodio, Switzerland), polypropylene (PP) membrane (Celgard 3501), 18 μm of copper foil, and coin-type cell (CR2032) assembly packages were purchased from Wellcos Co. (Gunpo, Republic of Korea). A lithium metal chip was purchased from MTI Korea (Seoul, Republic of Korea).

### 2.2. Synthesis of Fe_3_O_4_ NPs@rGO Composite

Fe_3_O_4_ NPs and Fe_3_O_4_ NPs@rGO were synthesized through a decomposition reaction of iron(III) acetylacetonate (Fe(acac)_3_) in the presence of a little Deionized (DI) water, so we first prepared Fe(acac)_3_ precursors. In a round-bottom flask, 32.44 g of FeCl_3_ (0.20 mol, 1.00 equiv.) was dissolved into 200 mL of DI water. Then, 70 mL of pentane-2,4-dione (0.68 mol, 3.41 equiv.) was subsequently added to a reaction pot. After stirring for one hour, 120 mL of triethylamine was poured into a reaction mixture and vigorously agitated overnight. The produced precipitations were collected by vacuum filtration and washed with DI water and a little cold ethanol. After vacuum-dried at 80 °C overnight, the obtained red powder has well-matched XRD patterns to Fe(acac)_3_ (Joint Committee on Powder Diffraction Standards (JCPDS) No. 30-1763) with a yield of 90% (63.72 g) [13]. For the synthesis of Fe_3_O_4_ NPs@rGO, 20 mL of oleylamine, where rGO powder was dispersed, was poured into a flame-dried, two-necked Schlenk flask equipped with a condenser. This suspension was heated to 120 °C for one hour under vacuum condition to remove residue water from the oleylamine surfactant. After the as-prepared 750 mg of Fe(acac)_3_ and 150 μL of DI water were added into a reaction mixture, the solution was kept at 290 °C for 3 h with an agitation under reflux. The precipitates were collected by centrifugation and washed with ethanol and n-hexane. Finally, black powder was obtained after vacuum-drying. For Fe_3_O_4_ NPs@rGO samples, the amount of powder added during the above synthesis process is indicated in parentheses. (e.g., Fe_3_O_4_ NPs@rGO (60 mg)) For comparison of electrochemical properties, brown-colored bare Fe_3_O_4_ NPs powder was also synthesized using the above procedure in the absence of an rGO template.

### 2.3. Characterization

Transmission electron microscopy (TEM) measurements were conducted using an FEI TECNAI TF30ST at an acceleration voltage of 300 kV, while scanning electron microscopy measurements were conducted using a JEOL JSM-7610F (Tokyo, Japan). X-ray diffraction (XRD) analysis was conducted using a Rigaku MiniFlex 600 (Tokyo, Japan) (Cu Kα radiation λ = 1.54051 Å, 40 kV, 15 mA). Raman spectra were measured using a LabRAM HR Evolution spectrometer (Kyoto, Japan) (λ = 514 nm). Thermogravimetric (TG) curves were obtained using a Thermo plus evo II at the heating rate of 10 °C min^−1^ under an air atmosphere. X-ray photoelectron spectroscopy (XPS) was performed using a Thermo VG Scientific Sigma Probe spectrometer (Waltham, MA, USA) (monochromatic Al Ka radiation, hν = 1486.6 eV). All electrochemical analyses were performed within a potential window of 0.01–3.0 V vs. Li/Li^+^. Cyclic Voltammetry (CV) and Galvanostatic charge-discharge cycle tests were conducted using a WonAtech WBCS3000S (Seoul, Republic of Korea). Electrochemical impedance spectroscopy (EIS) was performed using a WonAtech ZIVE SP1 over a frequency range of 10 mHz to 100 kHz.

### 2.4. Coin-Type (CR2032) Lithium Half-Cell Assembly

For the electrochemical analysis of the prepared Fe_3_O_4_ NPs and Fe_3_O_4_ NPs@rGO, 80 mg of one of them as an active material, 10 mg of Super P, and 10 mg of PVDF (polyvinylidene fluoride) were mixed in an agate mortar to be homogeneous ink. After coating on copper foil using a doctor blade, it was dried at 80 °C in air for one hour and then under vacuum for 3 h. The average active mass loadings of Fe_3_O_4_ NPs anode and Fe_3_O_4_ NPs@rGO anode were 1.0 and 0.3 mg cm^–1^, respectively. The as-prepared electrode was made into a 10 mm diameter disk and used as the working electrode. Lithium foil, a Celgard 3501 PP membrane, and 1 M LiPF_6_ in a solution of ethylene carbonate (EC) and dimethyl carbonate (DMC) (EC:DMC volume ratio = 1:1) were used as the counter-reference electrode, separator and electrolyte, respectively. All CR2032 lithium half-cells were fabricated in a pure argon gas purged glove box. (O_2_ and H_2_O < 0.1 ppm).

### 2.5. Electrochemical Analysis


(2) $$Z_{Re}=R_{s}+R_{ct}+{\sigma \omega }^{-0.5}$$

Z_Re_, R_s_, R_ct_, σ, and ω represent the real part of resistance, solution resistance, charge transfer resistance, Warburg coefficient, and angular frequency, respectively.

(3) $$D_{Li+}=\frac{R^{2}T^{2}}{2A^{2}n^{2}F^{4}C^{2}\sigma^{2}}$$

D_Li+_, R, T, A, n, F, and C are the diffusion coefficient of Li^+^ ions, gas constant, absolute temperature, area of electrode, number of transferred electrons, Faraday constant, and concentration of electrolyte.

(4) $$i={av}^{b}$$

(5) logi=loga+blogv

i and v are the current and scan rate.

(6) $$i_{t}=k_{1}v+k_{2}v^{0.5}$$

i_t_, k_1_v, and k_2_v^0.5^ denote the total current, capacitive current, and diffusion-controlled current.

## 3. Results and Discussion

**Figure 1:**
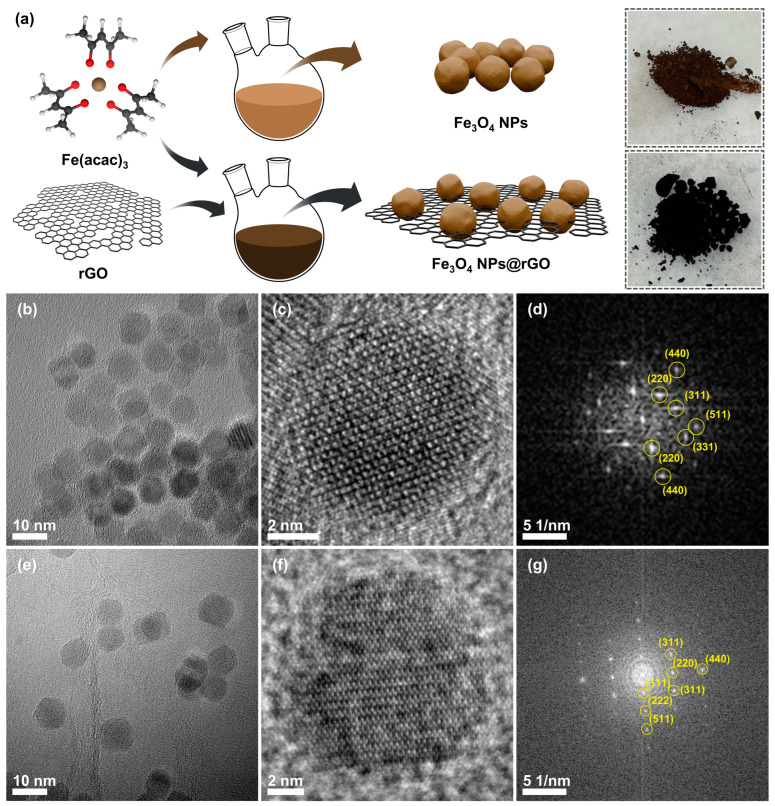
(**a**) Illustration of the one-pot synthetic process for Fe_3_O_4_ NPs and Fe_3_O_4_ NPs@rGO composite. Photo images: Fe_3_O_4_ NPs (top) and Fe_3_O_4_ NPs@rGO (60 mg) (bottom). (**b**,**e**) TEM images, (**c**,**f**) HR-TEM images, and (**d**,**g**) their corresponding FFT patterns of Fe_3_O_4_ NPs and Fe_3_O_4_ NPs@rGO, respectively. (**b**–**d**) Fe_3_O_4_ NPs, (**e**–**g**) Fe_3_O_4_ NPs@rGO.

Figure 1a shows the synthetic route for Fe₃O₄ NPs@rGO. In this process, the as-prepared iron(III) acetylacetonate, which already contains Fe–O bonds, reacts with a few drops of DI water in rGO-dispersed oleylamine, yielding Fe_3_O_4_ NPs@rGO composite materials via a one-pot colloidal synthesis. Conversely, when the rGO powder was omitted from the process, the final product was bare Fe_3_O_4_ NPs. Prepared Fe_3_O_4_ NPs and Fe₃O₄ NPs@rGO (60 mg) powders were observed by TEM and SEM analyses, revealing nanoparticles with a diameter of approximately 9 nm, as shown in Figure 1b,c,e,f. In addition, these nanoparticles in Fe_3_O_4_ NPs@rGO (60 mg) were uniformly distributed on the rGO surface due to the presence of the rGO template (Figures S1b and S3), whereas, in the absence of rGO templates, aggregation occurred among the nanoparticles (Figures S1a and S2). Also, Figure 1d and Figure 1g show the fast Fourier-transform (FFT) patterns corresponding to Figure 1c and Figure 1f, respectively, where reciprocal lattice spots indicate that these nanoparticles were composed of crystalline magnetite Fe_3_O_4_.

**Figure 2:**
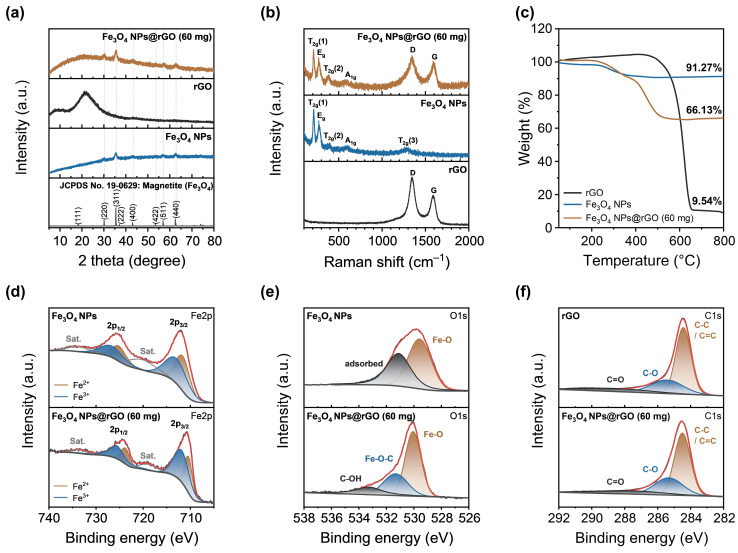
(**a**) XRD patterns, (**b**) Raman spectra, and (**c**) TGA curves of commercial rGO, Fe_3_O_4_ NPs, and Fe_3_O_4_ NPs@rGO (60 mg), respectively. XPS spectra of Fe_3_O_4_ NPs and Fe_3_O_4_ NPs@rGO (60 mg): (**d**) Fe2p and (**e**) O1s. (**f**) C1s XPS spectra of commercial rGO and Fe_3_O_4_ NPs@rGO (60 mg).

Figure 2a depicts the XRD patterns of commercial rGO, bare Fe_3_O_4_ NPs, and the Fe_3_O_4_ NPs@rGO (60 mg) composite. For bare Fe_3_O_4_ NPs, characteristic diffraction peaks were found at 30.15°, 35.5°, 43.17°, 57.07°, and 62.7°. Similarly, these peaks also appeared at 30.17°, 35.55°, 43.18°, 57.24°, and 62.83° in the Fe_3_O_4_ NPs@rGO (60 mg) composite powder. Each diffraction peak of both Fe_3_O_4_ and Fe_3_O_4_ NPs@rGO (60 mg) corresponded to (220), (311), (400), (422), (511), and (440) planes, respectively, suggesting that both bare Fe_3_O_4_ NPs and the Fe_3_O_4_ NPs@rGO (60 mg) composite have the crystalline structure of magnetite Fe_3_O_4_ (JCPDS No. 19-0629). Meanwhile, a broad peak originating from the rGO template appeared at around 21° for Fe_3_O_4_ NPs@rGO (60 mg) powder.

Figure 2b shows the Raman spectra of commercial rGO, bare Fe_3_O_4_ NPs, and the Fe_3_O_4_ NPs@rGO (60 mg) composite. The Fe_3_O_4_ NPs exhibit characteristic peaks at 212.9, 270.6, 392.4, 583.5, and 1289.8 cm^−1^, indexed to T_2g_(1), E_g_, T_2g_(2), A_1g_, and T_2g_(3) modes, corresponding to the translation of FeO_4_, symmetric bending, asymmetric stretching, symmetric stretching, and second-order scattering of Fe–O, respectively [14,15]. For the commercial rGO powder, the Raman spectrum of a typical carbonaceous material was shown. After deconvolution (Figure S4), we characterized peaks corresponding to D*, D, D″, G, and D′ bands, observed at 1231.2, 1346.7, 1518.6, 1581.7, and 1606.9 cm^−1^, respectively [16]. For the Fe_3_O_4_ NPs@rGO (60 mg) composite powder, the Raman spectrum displays peaks corresponding to T_2g_(1), E_g_, T_2g_(2), and A_1g_ modes of Fe_3_O_4_ NPs at 211.1, 269.5, 386.2, and 582.0 cm^−1^, respectively. Notably, after spectrum separation, the T_2g_(3) peak of Fe_3_O_4_ appeared at 1270.4 cm^−1^, and the peaks corresponding to D*, D, D″, G, and D′ bands derived from the rGO template were also independently found at 1236.7, 1346.6, 1502.1, 1585.5, and 1602.7 cm^−1^ (Figure S4). These results indicate that the prepared Fe_3_O_4_ NPs@rGO (60 mg) composite contains both Fe_3_O_4_ NPs and the rGO matrix together without any chemical changes.

As shown in Figure 2c, thermogravimetric (TG) analysis was conducted on commercial rGO, Fe_3_O_4_ NPs, and Fe_3_O_4_ NPs@rGO (60 mg) to determine the composite ratio of Fe_3_O_4_ NPs deposited on the rGO template in the Fe_3_O_4_ NPs@rGO (60 mg) sample. An expanded view on the low temperature region from 100 to 450 °C is illustrated in Figure S5. Both Fe_3_O_4_ and Fe_3_O_4_ NPs@rGO (60 mg) samples showed different extents of weight loss but similar trends with respect to temperature. A slight weight loss from room temperature to 150 °C indicated the removal of adsorbed water and volatile organic solvents. The bump appearing at 210 °C corresponds to the thermal decomposition to the Fe_2_O_3_ phase [17,18]. The weight decrease from 330 and 400 °C is thought to result from the evaporation of the oleylamine surfactant, which has a boiling point of 364 °C. Subsequently, a significant weight loss from 400 to 500 °C is attributed to the decomposition of the rGO component. Thus, the final phase at 800 °C for Fe_3_O_4_ NPs@rGO (60 mg) sample is considered to be Fe_2_O_3_ before calculating of the composition ratio for the Fe_3_O_4_ NPs@rGO (60 mg) sample. As the remaining weights at 800 °C for commercial rGO, Fe_3_O_4_ NPs, and Fe_3_O_4_ NPs@rGO (60 mg) were 9.54%, 91.27%, and 66.13%, respectively, the resulting composite ratio of Fe_3_O_4_ NPs in the Fe_3_O_4_ NPs@rGO (60 mg) sample was 63.92%.

Figure 2d–f are the XPS spectra corresponding to Fe2p, O1s, and C1s region. In the Fe2p XPS spectra (Figure 2d), some characteristic peaks appeared due to the spin–orbit splitting and complex chemical states of Fe_3_O_4_ compound, including Fe^2+^ from FeO and Fe^3+^ from Fe_2_O_3_. For Fe_3_O_4_ NPs (60 mg), these peaks were observed at 711.6, 712.9, 725.1, and 726.6 eV. The Fe_3_O_4_ NPs@rGO (60 mg) composite also revealed these peaks at 710.4, 711.8, 723.8, and 725.6 eV, which corresponded to Fe^2+^ 2p_3/2_, Fe^3+^ 2p_3/2_, Fe^2+^ 2p_1/2_, and Fe^3+^ 2p_1/2_, respectively. In the O1s region (Figure 2e), Fe_3_O_4_ NPs showed two typical characteristic peaks of Fe_3_O_4_ compound at 529.6 eV and 531.1 eV, corresponding to the Fe–O bond and adsorbed chemical species, including oxygen atom. For Fe_3_O_4_ NPs@rGO (60 mg), three peaks were observed at 530.1, 531.3, and 533.3 eV attributed to Fe–O, Fe–O–C, and C–OH bonds. The presence of a Fe–O–C bond suggested that the chemical interactions between Fe_3_O_4_ NPs and the rGO template occurred, and the C–OH bond originated from the rGO component. As shown in Figure 2f, the C1s XPS spectrum of Fe_3_O_4_ NPs@rGO (60 mg) was measured, because it contained rGO as a carbonaceous material. Thus, three peaks were located at 284.5, 285.3, and 287.7 eV, indicating C–C/C=C, C–O, and C=O bonds, respectively, originating from these bonds of commercial rGO component at 284.4, 285.4, 288.5 eV.

**Figure 3:**
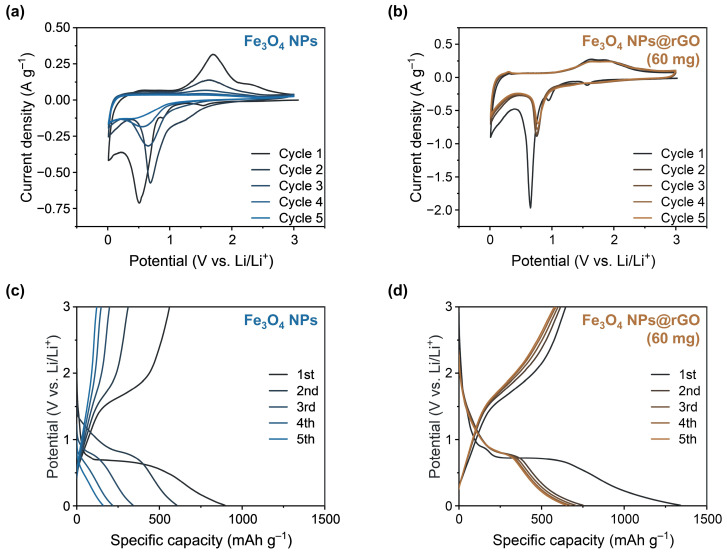
Initial 5 CV curves of (**a**) Fe_3_O_4_ NPs and (**b**) Fe_3_O_4_ NPs@rGO (60 mg) anode at a scan rate of 0.1 mV s^−1^. Galvanostatic charge–discharge profiles of (**c**) Fe_3_O_4_ NPs and (**d**) Fe_3_O_4_ NPs@rGO (60 mg) anodes at a current density of 1.0 A g^−1^.

To investigate the Li^+^ ion storage ability of Fe_3_O_4_ NPs and Fe_3_O_4_ NPs@rGO (60 mg), various electrochemical measurements were conducted in a coin-type (CR2032) lithium half-cell. Figure 3a,b are the initial five cyclic voltammograms of Fe_3_O_4_ NPs and Fe_3_O_4_ NPs@rGO (60 mg) at 0.1 mV s^−1^, respectively. For the bare Fe_3_O_4_ NPs anode, there were three peaks at 1.54, 0.89, and 0.51 V during the cathodic process, where each peak corresponded to the formation of Li_x_Fe_3_O_4_ through the insertion of Li^+^ ions into the crystalline lattice of Fe_3_O_4_ (Equation (7)), the formation of the solid electrolyte interface (SEI) layer due to the decomposition of electrolyte molecules on the anode surface, and the reductive conversion reaction by forming the metallic Fe^0^ and amorphous Li_2_O (Equation (8)), respectively. The large peak at 0.51 V shifted to 0.69 V over multiple cycles of CV test due to a loss of polarization. During the anodic process, there was the only one distinct peak at 1.69 V attributed to the oxidation of metallic Fe^0^ into Fe^2+^ and Fe^3+^ species (Equation (9)) [11].

(7) $${Fe}_{3}O_{4}+x{Li}^{+}+{xe}^{-}\to {Li}_{x}{Fe}_{3}O_{4}$$

(8) $${Li}_{x}{Fe}_{3}O_{4}+\left(8-x\right){Li}^{+}+{\left(8-x\right)e}^{-}\to {3Fe}^{0}+4{Li}_{2}O$$

(9) $${3Fe}^{0}+4{Li}_{2}O\to {Fe}_{3}O_{4}+{8Li}^{+}+{8e}^{-}$$

Likewise, the Fe_3_O_4_ NPs@rGO (60 mg) anode also showed three reduction peaks at 1.57, 0.95, and 0.65 V in the first cathodic sweep, corresponding to the formation of Li_x_Fe_3_O_4_, the formation of SEI film, and the conversion reaction, respectively. The reduction peak at 0.65 V of the first cathodic curve was also shifted to 0.78 V from the second CV cycle. During the anodic process, two peaks were observed at 1.64 and 1.90 V, corresponding to the oxidation of metallic Fe^0^ into Fe^2+^ and Fe^3+^ species, where these peaks appeared distinctly separated, unlike those of the Fe_3_O_4_ NPs anode. In this section, the CV analysis suggested poor electrochemical reversibility for the Fe_3_O_4_ NPs with disappearance of redox peaks, whereas the Fe_3_O_4_ NPs@rGO (60 mg) anode exhibited powerful electrochemical reversibility for the insertion and extraction of Li^+^ ions into the anode material.

Figure 3c,d illustrate the galvanostatic charge and discharge cycle profiles of Fe_3_O_4_ NPs and Fe_3_O_4_ NPs@rGO (60 mg) at a current density of 1.0 A g^−1^ during the initial five cycles, respectively. For the bare Fe_3_O_4_ NPs anode, electrochemical reversibility distinctly disappeared within the initial five cycles, like its cyclic voltammograms. Charge and discharge capacities corresponding to each cycle number of Fe_3_O_4_ NPs anode were as follows: (1st, 561 and 896 mAh g^−1^), (2nd, 311 and 606 mAh g^−1^), (3rd, 199 and 342 mAh g^−1^), (4th, 148 and 217 mAh g^−1^), and (5th, 121 and 160 mAh g^−1^). On the other hand, the Fe_3_O_4_ NPs@rGO (60 mg) anode exhibited excellent reversible capacity for Li^+^ ions storage, with specific capacities as follows: (1st, 646 and 1341 mAh g^−1^), (2nd, 615 and 752 mAh g^−1^), (3rd, 599 and 705 mAh g^−1^), (4th, 587 and 673 mAh g^−1^), and (5th, 577 and 651 mAh g^−1^). 

**Figure 4:**
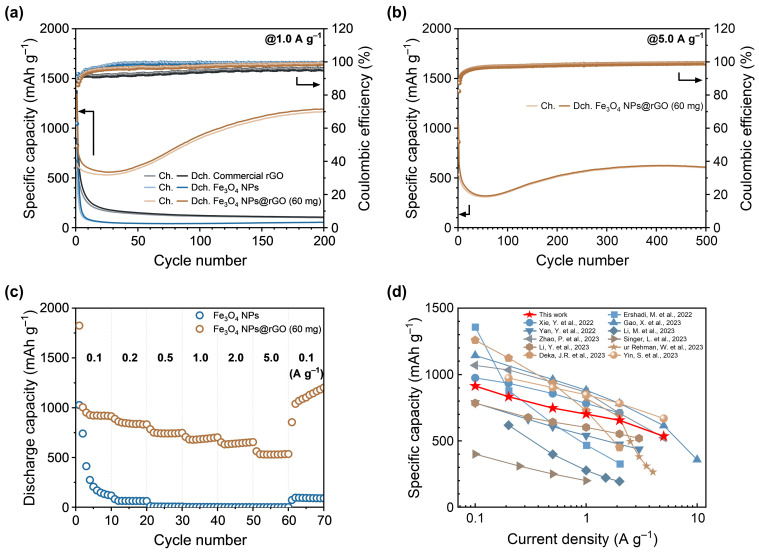
(**a**) Cycle performance of commercial rGO, Fe_3_O_4_ NPs, and Fe_3_O_4_ NPs@rGO (60 mg) anodes at a current density of 1.0 A g^−1^. (**b**) Cycle performance of Fe_3_O_4_ NPs@rGO (60 mg) anode at a current density of 5.0 A g^−1^. (**c**) Rate capability of Fe_3_O_4_ NPs and Fe_3_O_4_ NPs@rGO (60 mg) anodes at various current densities. (**d**) Comparison for rate performance of Fe_3_O_4_ NPs@rGO (60 mg) anode with literature data in Fe_3_O_4_-based anode materials [4,9,10,11,19,21,22,23,24,25,26].

Figure 4a shows the cycle performance of commercial rGO, Fe_3_O_4_ NPs, and Fe_3_O_4_ NPs@rGO (60 mg) anodes at a current density of 1.0 A g^−1^. The Fe_3_O_4_ NPs anode showed drastic degradation of specific capacity to below 100 mAh g^−1^ within the initial 10 cycles, whereas the Fe_3_O_4_ NPs@rGO revealed an improved cycle stability, with charge and discharge capacities of 1165 and 1191 mAh g^−1^ over 200 cycles, respectively. Herein, the capacity degradation was observed during the first few cycles. It was induced by the irreversible SEI formation and incomplete conversion reaction between metallic Fe^0^ and Fe_3_O_4_. And then, the capacity increase occurred after a decrease, which is known to be caused by the formation and decomposition of a reversible SEI layer related to the lithium-containing gel-like polymer film [7,11,19,20].

To examine the contribution of the Fe_3_O_4_ NPs to the overall capacity of the Fe_3_O_4_ NPs@rGO (60 mg) composite, galvanostatic charge–discharge cycle test was performed at 1.0 A g^−1^ for the commercial rGO anode. The Cycle performance (cycle number, charge, and discharge capacities) of commercial rGO anode over 200 cycles was as follows: (1st, 679 and 1376 mAh g^−1^), (2nd, 521 and 769 mAh g^−1^), and (200th, 100 and 105 mAh g^−1^). As the weight ratio of Fe_3_O_4_ NPs in Fe_3_O_4_ NPs@rGO (60 mg) composite was 63.92%, the average capacity contribution of Fe_3_O_4_ NPs component during 200 cycles was 93% (Figure S6). Thus, it is concluded that the Fe_3_O_4_ NPs deposited on the rGO template could be electrochemically activated, providing enhanced cycle performance. Moreover, additional composite samples were prepared with 30 mg or 90 mg of commercial rGO powder to evaluate their electrochemical properties and cycle performance (Figures S7 and S8). As a result, it was demonstrated that the optimized sample onto electrochemical properties was the Fe_3_O_4_ NPs@rGO (60 mg), revealing the best cycle performance in Figure S9. Meanwhile, it was investigated whether the treatment of commercial rGO with oleylamine surfactant would induce structural changes, such as N-doping modification of rGO matrix, and whether these changes could contribute to the enhancement of the electrochemical performance of Fe_3_O_4_ NPs@rGO composites. As described in the Supplementary Materials (Figures S10–S13), it was considered that the improved cycle performance of Fe_3_O_4_ NPs@rGO (60 mg) anode did not result from oleylamine treatment.

As shown in Figure 4b, the Fe_3_O_4_ NPs@rGO (60 mg) anode also exhibited excellent fast charge–discharge cycle stability. At 5.0 A g^−1^, Fe_3_O_4_ NPs@rGO (60 mg) anode delivered the 1st charge and discharge capacities of 536 and 1038 mAh g^−1^ and the 2nd charge and discharge capacities of 524 and 651 mAh g^−1^. Charge and discharge capacities after 500 cycles were 603 and 608 mAh g^−1^, respectively. These results demonstrate that the introduction of rGO template allowed the deposited Fe_3_O_4_ NPs to store Li^+^ ions at a high current density, exhibiting an improved rate capability and fast charge and discharge stability.

Figure 4c illustrates the rate capability of Fe_3_O_4_ NPs and Fe_3_O_4_ NPs@rGO (60 mg) measured by repeating 10 charge–discharge cycles at different current densities from 0.1 to 5.0 A g^−1^, which the current density was finally returned to 0.1 A g^−1^. The Fe_3_O_4_ NPs anode exhibited poor rate capability due to its dismal cycle performance. On the other hand, the Fe_3_O_4_ NPs@rGO (60 mg) anode delivered the following 10th discharge capacities: 914, 833, 747, 702, 655, and 535 mAh g^−1^ at 0.1, 0.2, 0.5, 1.0, 2.0 and 5.0 A g^−1^, respectively. At the end, it showed 1202 mAh g^−1^ when current density became 0.1 A g^−1^ again. These results indicated that the Fe_3_O_4_ NPs@rGO (60 mg) anode in this work revealed excellent rate performance despite being synthesized via a simple one-pot synthesis strategy by the in situ growth of Fe_3_O_4_ NPs onto the rGO surface, which was superior or comparable to the previously reported Fe_3_O_4_-based anode materials in recent three years in Figure 4d [4,9,10,11,19,21,22,23,24,25,26].

**Figure 5:**
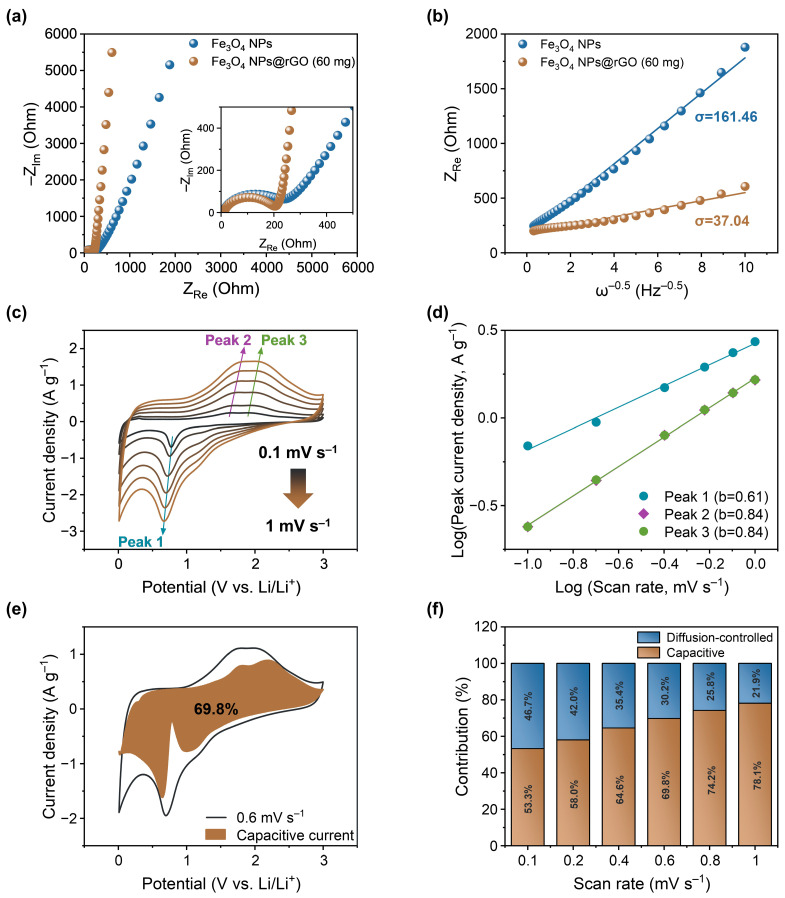
(**a**) Nyquist plots and (**b**) relationship between the real part of resistance and the inverse of square root of angular frequency of Fe_3_O_4_ NPs and Fe_3_O_4_ NPs@rGO (60 mg) anodes at OCV state. (**c**) Cyclic voltammograms of Fe_3_O_4_ NPs@rGO anode at various scan rates. (**d**) Relationship between logarithm of peak current density and the logarithm of scan rate for Peaks 1 to 3. (**e**) Visualized capacitive current component of Fe_3_O_4_ NPs@rGO (60 mg) anode at 0.6 mV s^−1^. (**f**) Ratio of diffusion-controlled current and capacitive current to total current at different scan rates of Fe_3_O_4_ NPs@rGO (60 mg) anode.

Figure 5 presents the results obtained from various electrochemical analyses using Equations (2)–(6) in the experimental section. Figure 5a illustrates the Nyquist plots of Fe_3_O_4_ NPs and Fe_3_O_4_ NPs@rGO (60 mg) anodes in the open-circuit voltage (OCV) state within the frequency range of 10 mHz to 100 kHz. The inset graph shows the expanded view of the −Z_Im_ and Z_Re_ range from 0 to 500 Ω, where the charge transfer resistances (R_ct_) were 239 and 203 Ω, respectively. When plotting the real part of resistance (Z_Re_) of Fe_3_O_4_ NPs and Fe_3_O_4_ NPs@rGO (60 mg) against the inverse of square root of angular frequency (ω) for Figure 5a, linear relationships were observed as shown in Figure 5b [27]. Herein, the slopes (σ) of each line for Fe_3_O_4_ NPs and Fe_3_O_4_ NPs@rGO (60 mg) anodes were 161.46 and 37.04 Ω s^−0.5^, and diffusion coefficient of Li^+^ ions (D_Li+_) could be obtained from these σ values [27]. The calculated D_Li+_ values of Fe_3_O_4_ NPs and Fe_3_O_4_ NPs@rGO (60 mg) anodes were 2.20 × 10^−12^ and 4.19 × 10^−11^, respectively. As a result, the improved R_ct_ and D_Li+_ values of Fe_3_O_4_ NPs@rGO (60 mg) anodes suggest that the rGO template, introduced by the one-pot synthesis composite strategy, enhanced the electronic and ionic conductivities of Fe_3_O_4_ NPs@rGO (60 mg) anode.

Figure 5c displays cyclic voltammograms at various scan rates of 0.1, 0.2, 0.4, 0.6, 0.8, and 1.0 mV s^−1^ to further study the electrochemical kinetics of Fe_3_O_4_ NPs@rGO (60 mg) anode. There was little polarization for Peaks 1, 2, and 3 as scan rates increased, and the linear relationships between the logarithm of peak current density for these peaks and the logarithm of scan rate are shown in Figure 5d. The slope of each line represents the b value [28], which determines its electrochemical property. Thus, peak 1 (b = 0.61) is indicative of a diffusion-controlled process, while peaks 2 and 3 (b = 0.84) suggest a capacitive process.

In addition, the electrochemical property of the Fe_3_O_4_ NPs@rGO (60 mg) anode was further studied by splitting the total current into diffusion-controlled current and capacitive current [29]. The capacitive current obtained at 0.6 mV s^−1^ is visualized in Figure 5e, and the contributions of both capacitive current and diffusion-controlled current at different scan rates are shown in Figure 5f. Herein, the Fe_3_O_4_ NPs@rGO (60 mg) anode exhibited dominant capacitive behavior. As a result, Li^+^ ions could be rapidly transferred to the surface of the Fe_3_O_4_ NPs@rGO (60 mg) anode via its capacitive Li^+^ ion storage process, contributing to its excellent rate performance at high current densities.

## 4. Conclusions

In summary, a promising anode material, Fe_3_O_4_ NPs@rGO, was synthesized to enhance performance of LIBs through a facile one-pot synthesis with in situ growth of Fe_3_O_4_ NPs onto rGO surfaces. In particular, the Fe_3_O_4_ NPs@rGO (60 mg) anode showed more impressive reversible capacity of 1191 mAh g^−1^ at a current density of 1.0 A g^−1^ after 200 cycles and a higher rate capability with 608 mAh g^−1^ after 500 cycles at a high current density of 5.0 A g^−1^, compared to the bare Fe_3_O_4_ NPs. These results are similar to or superior to the rate performance of a series of Fe_3_O_4_-based anode materials reported in the past three years. Therefore, the Fe_3_O_4_@rGO (60 mg) showing a good rate performance can be easily prepared via a one-pot synthesis strategy without the need for burdensome processes, which increases its practical value. Furthermore, various electrochemical measurements demonstrated that the improved rate performance and excellent cycle stability are caused by enhanced electronic and ionic conductivities. In conclusion, this Fe_3_O_4_ NPs@rGO (60 mg) composite anode demonstrated high-rate capability and excellent fast-chargeable properties, making it a suitable material for advanced LIBs.

## Data Availability

The original contributions presented in the study are included in the article and Supplementary Material, further inquiries can be directed to the corresponding authors.

## Supplementary Materials

The following supporting information can be downloaded at: https://www.mdpi.com/article/10.3390/ma17205059/s1, Figure S1. Low-magnification TEM image of (a) Fe_3_O_4_ NPs and (b) Fe_3_O_4_ NPs@rGO (60 mg); Figure S2. SEM images at ×160k and ×60k magnifications for bare Fe_3_O_4_ NPs; Figure S3. SEM images at ×160k and ×60k magnifications for Fe_3_O_4_ NPs@rGO (60 mg) composite; Figure S4. Separated Raman spectra of commercial rGO, Fe_3_O_4_ NPs and Fe_3_O_4_ NPs@rGO (60 mg) powders in detail; Figure S5. Expanded view of TGA curves of commercial rGO, Fe_3_O_4_ NPs and Fe_3_O_4_ NPs@rGO (60 mg) powders from 100 to 450 °C; Figure S6. Capacity contributions derived from Fe_3_O_4_ component of Fe_3_O_4_ NPs@rGO (60 mg) anode at a current density of 1.0 A g^−1^ for 200 cycles; Figure S7. SEM images at ×160k and ×60k magnifications for Fe_3_O_4_ NPs@rGO (30 mg) composite; Figure S8. SEM images at ×160k and ×60k magnifications for Fe_3_O_4_ NPs@rGO (90 mg) composite; Figure S9. Initial 5 CV curves of (a) Fe_3_O_4_ NPs@rGO (30 mg) and (b) Fe_3_O_4_ NPs@rGO (90 mg) composite anode at a scan rate of 0.1 mV s^−1^, respectively. Comparisons of cycle performance of Fe_3_O_4_ NPs@rGO (30, 60 and 90 mg) at (c) 1.0 and (d) 5.0 A g^−1^; Figure S10. SEM images of (a) commercial rGO and (b) OAm-rGO at ×20k magnification; Figure S11. XRD patterns of commercial rGO and OAm-rGO; Figure S12. XPS spectra of commercial rGO and OAm-rGO: (a) C1s, (b) O1s and (c) N1s; Figure S13. Cycle performance of commercial rGO and OAm-rGO at 1.0 A g^−1^.
