# Supplementary material for: Facile One-Pot Synthesis of Fe3O4 Nanoparticles Composited with Reduced Graphene Oxide as Fast-Chargeable Anode Material for Lithium-Ion Batteries
Source: Materials (Basel). 2024 Oct 17;17(20):5059. doi: 10.3390/ma17205059 (PMC11509682; doi:10.3390/ma17205059)
Supplement: Supplementary file 1 [file materials-17-05059-s001.zip › materials-3238710-supplementary.pdf]

Supplementary Materials

Facile One-pot Synthesis of Fe_3O_4 Nanoparticles Composited with Reduced Graphene Oxide as Fast-chargeable Anode Material for Lithium-ion Batteries

Honggyu Seong [1,†], Taejung Jung [1,†], Sanghyeon Kim [2,*] and Jaewon Choi [1,*]

[1] Department of Chemistry and Research Institute of Molecular Alchemy, Gyeongsang National University, Jinju 52828, Republic of Korea.

[2] Department of Materials Science and Engineering, Inha University, Incheon 22212, Republic of Korea.

* Correspondence: skim07@inha.ac.kr (S.K); cjw0910@gnu.ac.kr (J.C.)

† These authors contributed equally to this work.

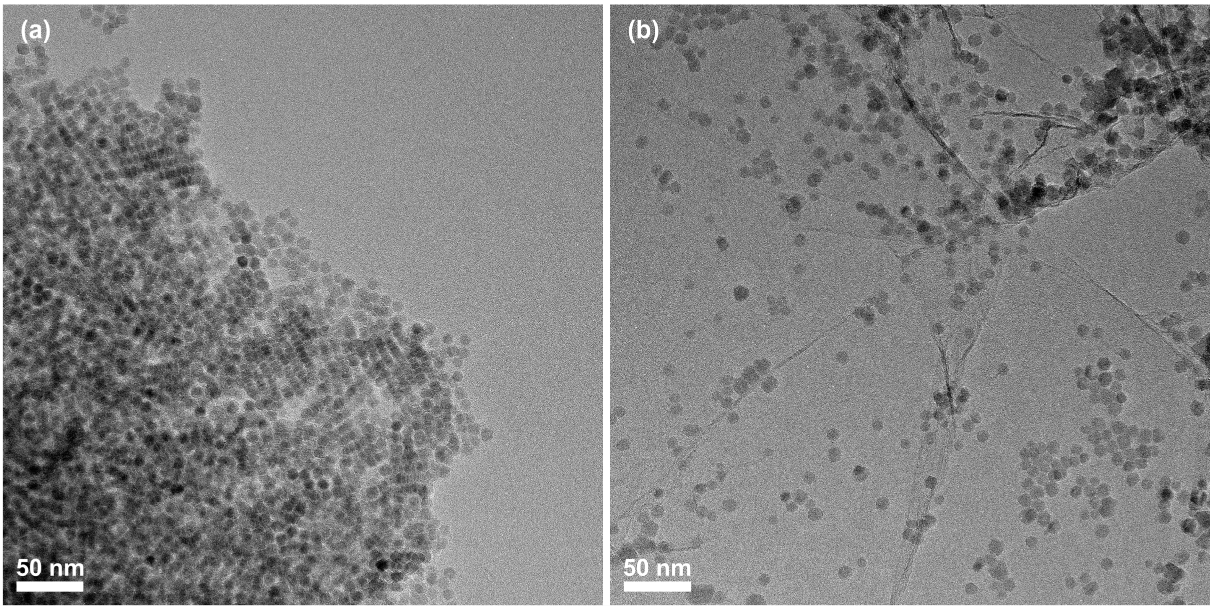

Figure S1. Low-magnification TEM image of (**a**) Fe_3O_4 NPs and (**b**) Fe_3O_4 NPs@rGO (60 mg).

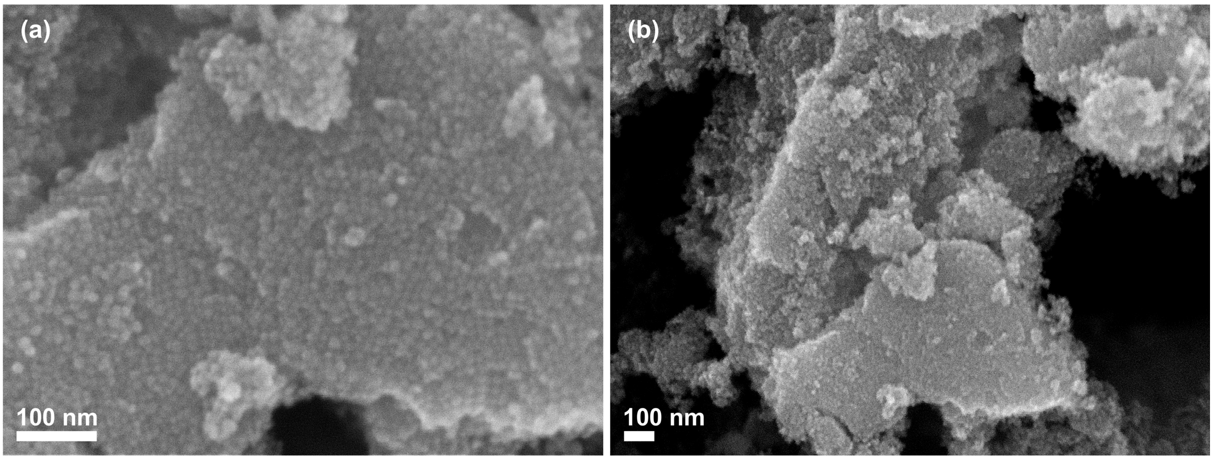

Figure S2. SEM images at ×160k and ×60k magnifications for bare Fe_3O_4 NPs.

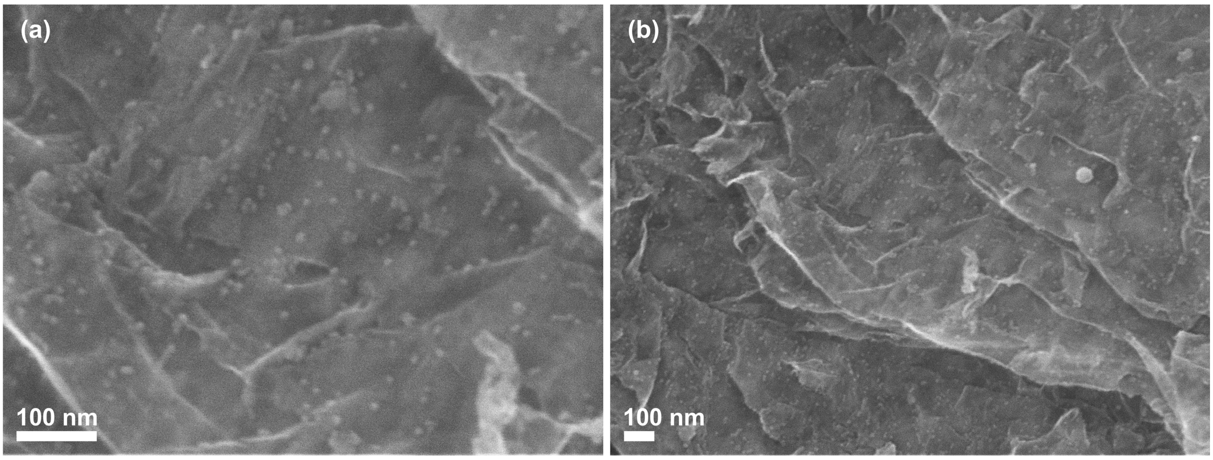

Figure S3. SEM images at ×160k and ×60k magnifications for Fe_3O_4 NPs@rGO (60 mg) composite.

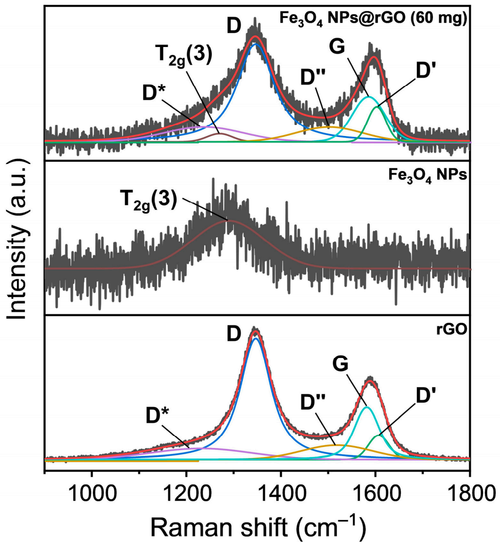

Figure S4. Separated Raman spectra of commercial rGO, Fe_3O_4 NPs and Fe_3O_4 NPs@rGO (60 mg) powders in detail.

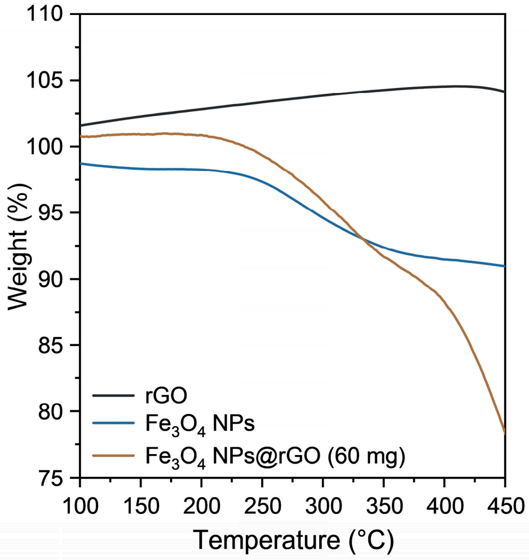

Figure S5. Expanded view of TGA curves of commercial rGO, Fe_3O_4 NPs and Fe_3O_4 NPs@rGO (60 mg) powders from 100 to 450 °C.

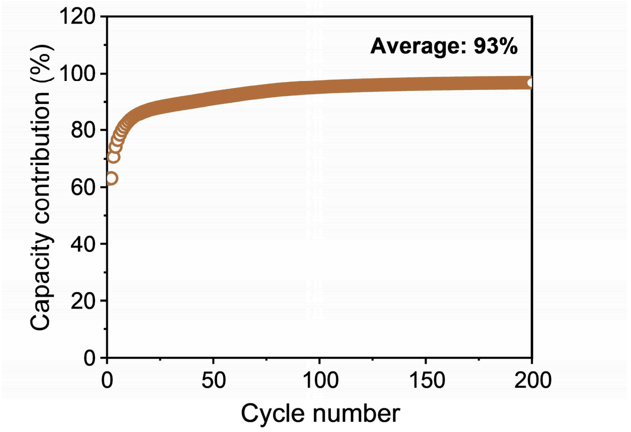

Figure S6. Capacity contributions derived from Fe_3O_4 component of Fe_3O_4 NPs@rGO (60 mg) anode at a current density of 1.0 A g^{-1} for 200 cycles.

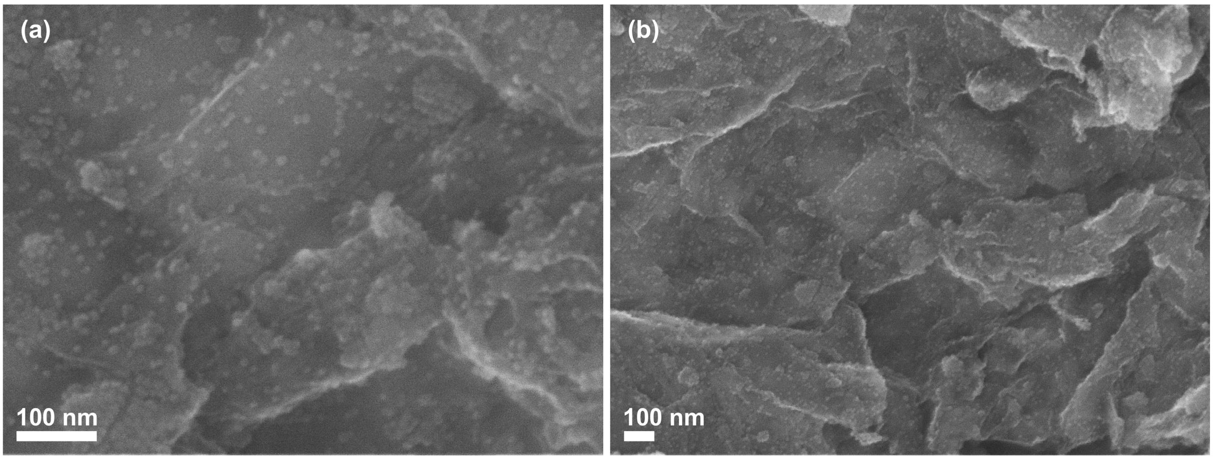

Figure S7. SEM images at ×160k and ×60k magnifications for Fe_3O_4 NPs@rGO (30 mg) composite.

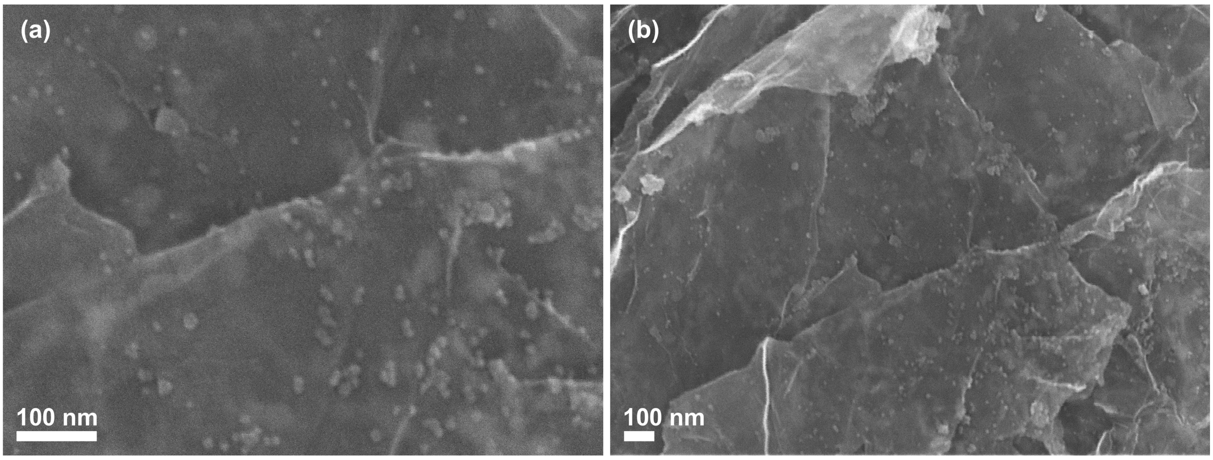

Figure S8. SEM images at ×160k and ×60k magnifications for Fe_3O_4 NPs@rGO (90 mg) composite.

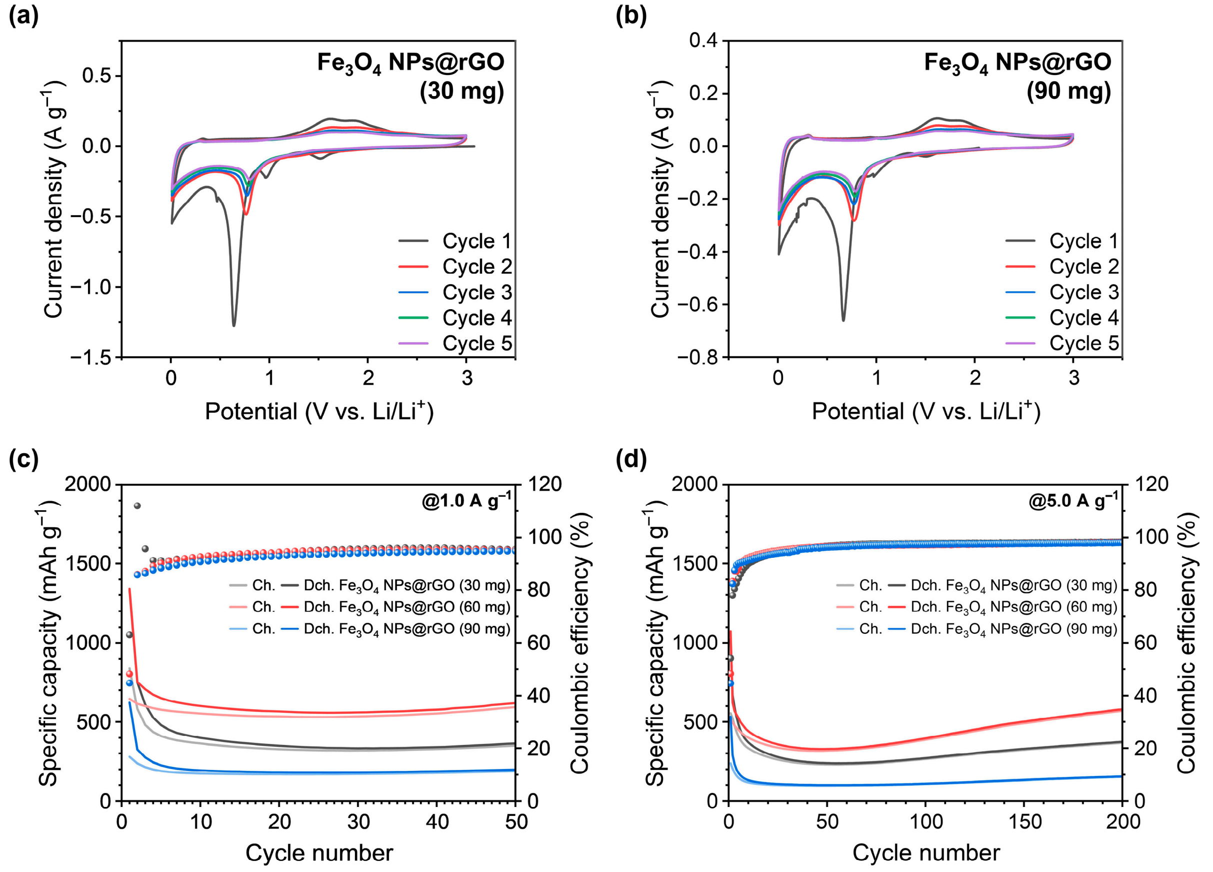

Figure S9. Initial 5 CV curves of (**a**) Fe_3O_4 NPs@rGO (30 mg) and (**b**) Fe_3O_4 NPs@rGO (90 mg) composite anode at a scan rate of 0.1 mV s^{-1}, respectively. Comparisons of cycle performances of Fe_3O_4 NPs@rGO (30, 60 and 90 mg) at (**c**) 1.0 and (**d**) 5.0 A g^{-1}.

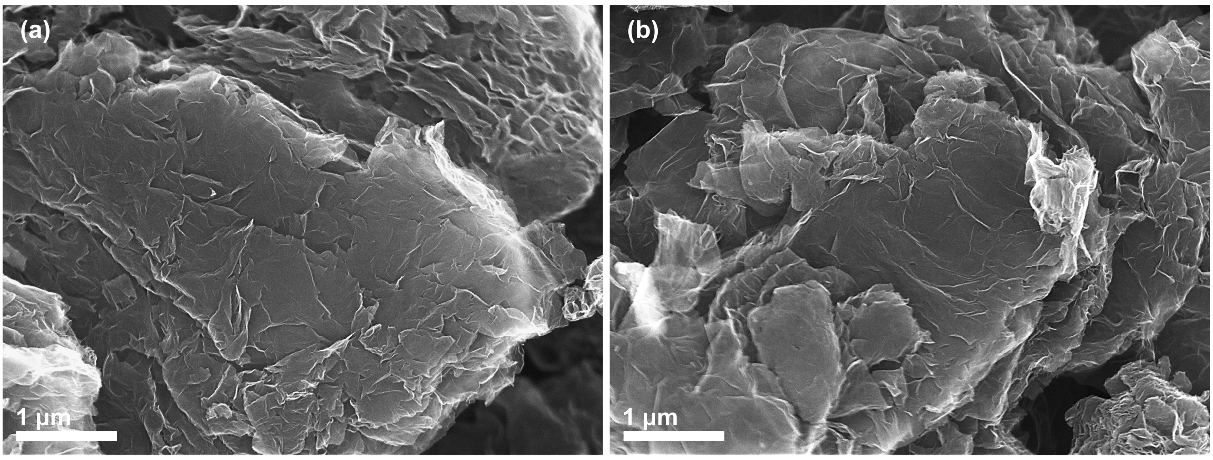

Figure S10. SEM images of (**a**) commercial rGO and (**b**) OAm-rGO at ×20k magnification.

We investigated whether oleylamine surfactant in this work induces meaningful changes like N-doping modification. When observing the morphology of commercial rGO and oleylamine-treated rGO (denoted as OAm-rGO) using SEM analysis, the sheet-like morphology derived from commercial rGO was shown even after the oleylamine-treatment.

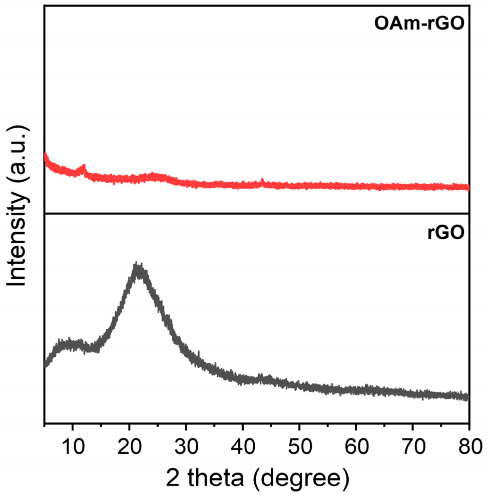

Figure S11. XRD patterns of commercial rGO and OAm-rGO.

However, the XRD pattern of OAm-rGO showed an unexpected result, which its characteristic peak almost disappeared and was slightly shifted from around 21° to around 24°. The crystallinity of OAm-rGO indicated evidently amorphous phase with a disorder and narrow interlayer distance compared to commercial rGO.

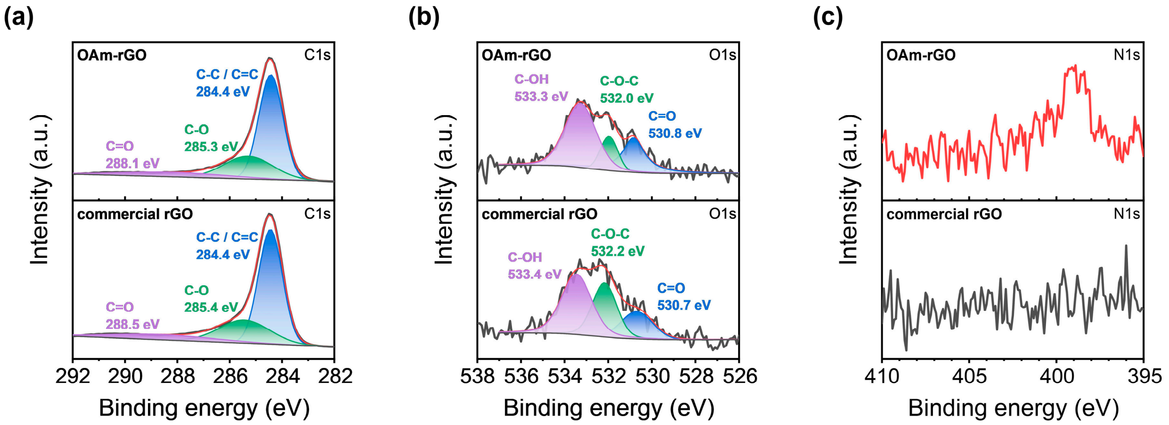

Figure S12. XPS spectra of commercial rGO and OAm-rGO: (**a**) C1s, (**b**) O1s and (**c**) N1s.

For a result of XPS analysis, C1s and O1s XPS spectra showed that commercial rGO and OAm-rGO had the similar chemical states for carbon and oxygen species, respectively. Likewise, as shown in Figure S4, Raman spectra of Fe_3O_4 NPs@rGO (60 mg) and commercial rGO were also similar in the range from 800 to 1800 cm^{-1}. These results indicated that the carbonaceous structure from commercial rGO template was not changed, because the synthesis temperature of 290°C in this work was too mild for nitrogen atom to penetrate into the chemical structure of carbonaceous rGO. Though it all, N1s spectrum of OAm-rGO displayed a small peak at around 399 eV, indicating the presence of nitrogen species that commercial rGO did not have. Therefore, the nitrogen species corresponding to this new peak in the N1s XPS spectrum of OAm-rGO sample was expected to be derived from trace amounts of adsorbed oleylamine that was not completely removed during the washing process.

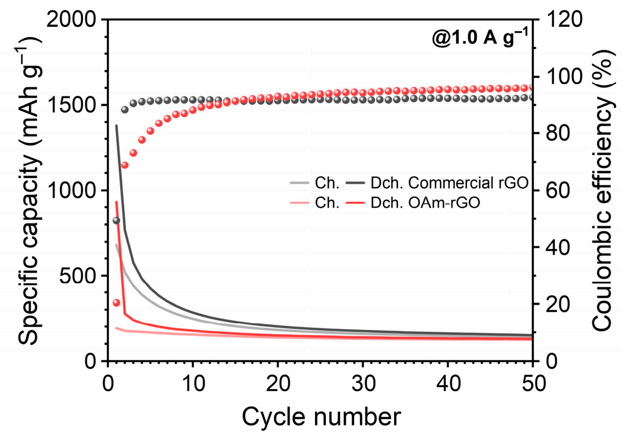

Figure S13. Cycle performances of commercial rGO and OAm-rGO at 1.0 A g^{-1}.

For the galvanostatic charge–discharge cycle tests, the OAm-rGO anode rather delivered a lower reversible capacity than the commercial rGO anode. Thus, it is difficult to consider OAm-rGO sample as a N-doped carbonaceous material with better electrochemical properties. Since the cycle performances of the OAm-rGO was degraded than that of the commercial rGO after heating in oleylamine surfactant, it is believed that an insulation by organic alkyl chains of residual oleylamine rather than the N-doping modification of commercial rGO occurred during the in-situ growth process in oleylamine surfactant.
